# Active recombinant *Tol2* transposase for gene transfer and gene discovery applications

**DOI:** 10.1186/s13100-016-0062-z

**Published:** 2016-03-31

**Authors:** Jun Ni, Kirk J. Wangensteen, David Nelsen, Darius Balciunas, Kimberly J. Skuster, Mark D. Urban, Stephen C. Ekker

**Affiliations:** Department of Biochemistry and Molecular Biology, Mayo Clinic, 200 1st St SW, 1342C Guggenheim, Rochester, MN 55905 USA; Department of Chemical and Systems Biology, Stanford University School of Medicine, Stanford, CA 94305 USA; Department of Biochemistry, Molecular Biology, and Biophysics, University of Minnesota, Minneapolis, MN 55455 USA; University of Pennsylvania, 9 Penn Tower, 3400 Spruce ST, Philadelphia, PA 19104 USA; Department of Biology, Temple University, Philadelphia, PA 19122 USA

**Keywords:** *Tol2* transposase, *hAT* superfamily, Recombinant transposase protein, Zebrafish, Transposition site preference

## Abstract

**Background:**

The revolutionary concept of “jumping genes” was conceived by McClintock in the late 1940s while studying the *Activator/Dissociation* (*Ac/Ds*) system in maize. Transposable elements (TEs) represent the most abundant component of many eukaryotic genomes. Mobile elements are a driving force of eukaryotic genome evolution. McClintock’s *Ac*, the autonomous element of the *Ac/Ds* system, together with *hobo* from *Drosophila* and *Tam3* from snapdragon define an ancient and diverse DNA transposon superfamily named *hAT.* Other members of the *hAT* superfamily include the insect element *Hermes* and *Tol2* from medaka. In recent years, genetic tools derived from the ‘cut’ and ‘paste’ *Tol2* DNA transposon have been widely used for genomic manipulation in zebrafish, mammals and in cells in vitro.

**Results:**

We report the purification of a functional recombinant *Tol2* protein from *E.coli*. We demonstrate here that following microinjection using a zebrafish embryo test system, purified *Tol2* transposase protein readily catalyzes gene transfer in both somatic and germline tissues in vivo. We show that purified *Tol2* transposase can promote both in vitro cutting and pasting in a defined system lacking other cellular factors. Notably, our analysis of *Tol2* transposition in vitro reveals that the target site preference observed for *Tol2* in complex host genomes is maintained using a simpler target plasmid test system, indicating that the primary sequence might encode intrinsic cues for transposon integration.

**Conclusions:**

This active *Tol2* protein is an important new tool for diverse applications including gene discovery and molecular medicine, as well as for the biochemical analysis of transposition and regulation of *hAT* transposon/genome interactions. The measurable but comparatively modest insertion site selection bias noted for *Tol2* is largely determined by the primary sequence encoded in the target sequence as assessed through studying *Tol2* protein-mediated transposition in a cell-free system.

**Electronic supplementary material:**

The online version of this article (doi:10.1186/s13100-016-0062-z) contains supplementary material, which is available to authorized users.

## Background

Our understanding of transposable elements (TEs) begins with McClintock’s revolutionary work with the *Activator/Dissociation* (*Ac/Ds*) system in maize [1]. *Ac*, the autonomous element of the *Ac/Ds* system, together with *hobo* from *Drosophila* and *Tam3* from snapdragon define an ancient and diverse DNA transposon superfamily named *hAT* [2–4]*.* Widespread in plants and animals, *hAT* transposons are the most abundant DNA transposons in humans [5]. However, none of the human *hAT* elements have been active during the past 50 million years [5].

The first active DNA transposon discovered in vertebrates was the medaka fish (*Oryzias latipes*)-derived *hAT* element *Tol2* [6]*. Tol2* shares a number of features with other *hAT* members including transposases with a DDE (aspartate-aspartate-glutamate) catalytic motif, short terminal inverted repeats (TIRs) and formation of 8-bp host duplications upon transposition [2, 7]. Because derivatives from *Tol2* have high cargo-capacity and low susceptibility to over-production inhibition, they have become popular gene transfer agents in a variety of animal systems, including zebrafish, African frog, chicken, mouse and human cell cultures including primary T cells [8], and for various genome biology applications (for reviews, see [9, 10].

TEs generally display very diverse patterns of target site selectivity. Studying the mechanisms for such selection is useful to gain insights on the biology of transposition, shedding light on the genome structure and designing better transposon tools for specific applications. Previous research has suggested that the mechanisms of target site selection are very complex and varied from mobile element to mobile element. In many cases, it involves the direct interaction between the transposase/recombinase and the target DNA or their indirect communication through accessory proteins [11]. However, specific factors that contribute to *hAT* element integration preference are largely unknown.

Our knowledge of transposition is largely based on bacterial TEs. Less is known about eukaryotic transposase proteins since they have been historically more difficult to express and reconstitute in vitro. In the present work, we establish a recombinant *Tol2* protein-based system to serve as a tool for genome engineering and to probe the transposition mechanism of this vertebrate *hAT* transposon, focusing on the integration steps. We directly compare known *Tol2* isoforms for activity in both human cells and zebrafish in vivo. We demonstrate that the highest activity variant can be epitope-tagged and retain full activity, and we purify epitope-tagged *Tol2* protein (*His-Tol2*) from E. coli. The functionality of this recombinant *Tol2* transposase is demonstrated in vivo in zebrafish using both somatic and germline transposition assays. Thus *His-Tol2* protein is a viable new source of transposase for molecular medicine and genome engineering applications.

We further show that purified *His-Tol2* can carry out both the excision and integration steps of transposition in vitro in the absence of any cellular co-factors. *Tol2* displays a modest preference for AT-rich DNA in vivo [12]. Such insertion bias is also noted in a cell-free and defined assay when the insertion distribution of *miniTol2* into a target plasmid was measured. *miniTol2* contains the transposon end sequences necessary and sufficient in vivo for excision and integration [13, 14]. *miniTol2* insertion is accompanied by 8-bp target site duplications as occurs in vivo and displays an insertion site preference in vitro, with a higher likelihood of insertion into AT-rich sequences similar to that noted for in vivo integrations [12]. These results suggest the target selection mechanism is at least in part maintained in this much simpler system.

## Results

### The 649 amino acid *Tol2* isoform is the most active transposase in vivo and in vitro

**Fig. 1:**
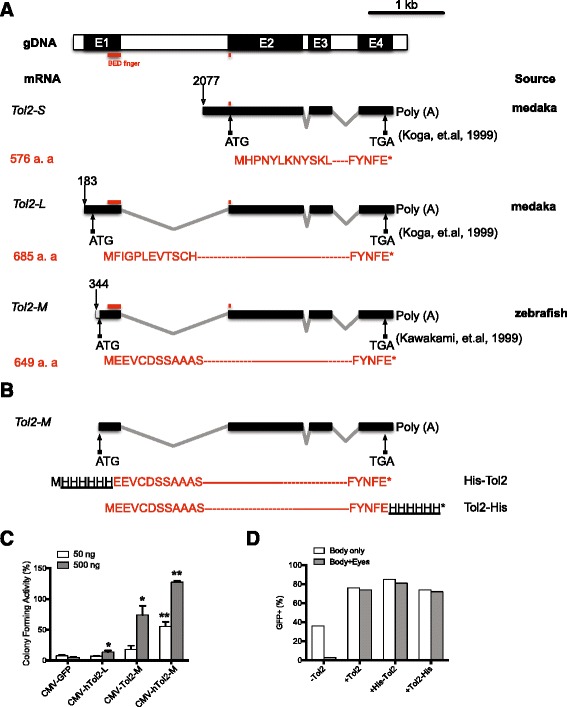
The highest activity *Tol2* isoform and its modification by 6X His tag. **a** *Tol2* genomic DNA (GenBank: D84375.2) structure and expressed mRNA from various sources (*Tol2-S* GenBank: AB031080; *Tol2-L* GenBank: AB031079; *Tol2-M* GenBank: AB032244). The first nucleotide location is numbered in relation to genomic DNA sequence in each mRNA and indicated by an open-ended arrow. The start and stop codons of the longest open reading frame in each mRNA are also indicated. *Tol2-L* spans all 4 exons (black bar, E1-4) while *Tol2-S* covers only the last three (E2-4). *Tol2-M* starts further into exon 1 than *L*. The predicted *Tol2-M* protein is slightly shorter at the amino-terminus than *Tol2-L*. Corresponding sequences encoding the BED zinc finger DNA binding motif are indicated on genomic DNA and mRNA. **b** In this paper we used a modified *Tol2-M* mRNA [13] encoding the same protein as *Tol2-M*, but all the nucleotides 5′ to the first start codon in *Tol2-M* (Fig. 1a, *Tol2-M* gray box) were deliberately removed to eliminate any possible effects from an out-of-frame ATG within those sequences. 6X His tags were added to either 5′- or 3′ of the modified mRNA, and the deduced N-terminus or C-terminus modified *Tol2* protein sequences were outlined. **c** Human codon-optimized *Tol2-M* was most effective for gene transfer in HeLa cells. *Tol2-M* was tested for activity in human cell culture by colony forming assay. A *Tol2* transposon vector carrying a Zeocin resistance gene cassette was co-transfected with different version of *Tol2* or GFP control driven by CMV promoter at 50 ng or 500 ng. The numbers of Zeocin-resistant colonies formed in each treatment were compared to the treatment with the highest colony numbers (500 ng CMV-hTo2-M) as percentages. Bars represent mean values ± SEM of three independent experiments. * or ** indicate data is significantly different from respective CMV-GFP controls (* *t*-test *P* < 0.05; ** *t*-test *P* < 0.01). **d** *Tol2* transposase could be modified by 6XHis epitope without loss of activity. A GFP-reporter [13] was co-injected with synthesized mRNA corresponding to untagged *Tol2* or *Tol2* tagged with 6X-His at either N-terminus (*His-Tol2*) or C-terminus (*Tol2-His*). Somatic GFP expression was scored at 3 dpf and the percentages of injected fish demonstrating GFP only in the body (body only) or demonstrating strong GFP in eyes and anywhere in the body (body + eyes) were recorded

Different coding sequences and activities have been described in previous works for the *hAT* transposase *Tol2*. Two distinct endogenous *Tol2* mRNAs were identified in the original medaka fish isolate [15]. The shorter mRNA (*Tol2-S*, GenBank accession number AB031080) is encoded by exons 2–4 and results in a predicted protein of 576 amino acids [15] (Fig. 1a). The longer *Tol2* mRNA (*Tol2-L*, GenBank accession number AB031079) is encoded by exons 1–4, and a protein of 685 amino acids is predicted using the first in-frame start codon [15] (Fig. 1a). *Tol2-S* inhibits excision by the *Tol2-L* protein, this inhibition was probably not through competition of DNA-binding but some other unknown mechanism, as *Tol2-S* protein lacks the majority of the BED zinc finger motif [16] (Fig. 1a). This also suggests that the sequences encoded by exon 1 are critical to *Tol2* transposase function. When a copy of medaka *Tol2* genomic sequence was introduced into zebrafish cells that do not harbor any endogenous *Tol2* elements, a third *Tol2* isoform of different length was identified (*Tol2-M*) [17] (Fig. 1a). This mRNA encodes a predicted protein of 649 amino acids that is shorter at the N-terminus than *Tol2-L* (Fig. 1a). This latter, heterologous mRNA (*Tol2-M*) is used in nearly all currently available *Tol2*-based genetic tools [13, 18–20]. The activity of the naturally occurring *Tol2-L* has not been explored in detail or compared to *Tol2-M* under similar conditions.

To determine whether the isoforms of *Tol2* might encode different functional outcomes, we compared *Tol2* isoforms in human HeLa cell transposition assays upon co-transfection of transposase helper and transposon donor plasmids [13]. For *Tol2-M*, the gene-transfer activity is positively related to the amount of *Tol2* transposase provided as plasmids (Fig. 1c). In addition, through codon optimization [21], we increased gene transfer in HeLa cells by ‘humanizing’ the *Tol2-M* codon usage (*hTol2-M*). The same strategy was applied to *Tol2-L*. However, even this ‘humanized’ *Tol2-L* (*hTol2-L*) showed reduced activity compared to *Tol2-M* in human cells (Fig. 1c). *Tol2-M* also showed higher activity than *Tol2-L* in zebrafish transposition assays [13] (data not shown). Thus the 649 amino acid *Tol2-M* isoform shows the highest activity of the three described protein forms (Fig. 1a), and will be referred to hereafter as *Tol2* unless otherwise stated.

### *Tol2* can be modified on either the N- or C- terminus by 6XHis and retain full activity

The ability to identify and readily purify a protein is critical for the development of a system for biochemical analysis. Targeted modification of vertebrate transposase enzymes has been a challenge in the field. The addition of new sequences normally results in either reduced or limited enzymatic activity, as was the case for *Sleeping Beauty*, the most studied vertebrate transposable element [22, 23] or with *Tol2* in prior studies [24, 25]. Here we chose a small 6X-His protein tag for use with *Tol2* (Fig. 1b).

To test if such modification will compromise transposase activity, zebrafish embryos were injected with synthetic *Tol2* transposase mRNA encoding *Tol2*, 5′-His (6X) tagged *Tol2*, or 3′-His (6X) tagged *Tol2* as well as with a transposon reporter vector [13]. Embryos were scored for GFP-positive cells or eyes with green fluorescence at 3 days post-fertilization (dpf) as described [13]. Visualization of net gene transfer into the eyes of injected zebrafish embryos is a convenient and robust assay system for determining *Tol2*-mediated transposition in somatic tissues [13]. Importantly, the 6X-His tagged versions of *Tol2* showed the same activity as the untagged *Tol2* proteins in this in vivo transposition assay (Fig. 1d).

### Purified *His-Tol2* from *E.coli*

**Fig. 2:**
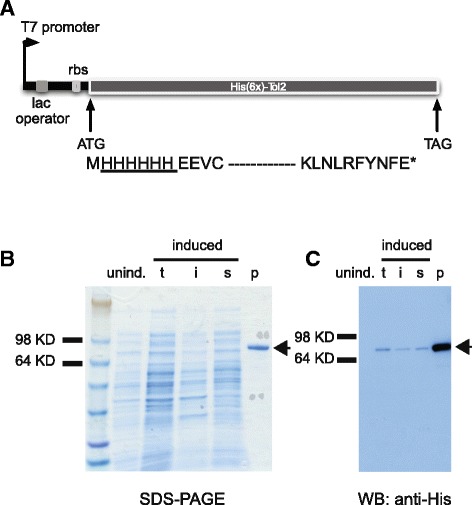
Expression and detection of recombinant *Tol2* transposase protein. **a** *Tol2* transpoase sequence was cloned into an N-terminal 6XHis expression vector pET-21a (Novagen). The expression cassette driven by T7 promoter is shown, and a fusion protein of ~74 kD was expected. rbs: ribosome binding site. **b** Expression of *His-Tol2* in *E.coli* BL21-AI strain. unind: cell lysate from uninduced cells harboring *Tol2*-expression vector; t: total induced crude cell lysate; i: insoluble protein fraction from induced cell lysate; s: soluble protein fraction from induced cell lysate. Equal volume of cell culture was loaded in each lane. p: ~ 350 ng purified *His-Tol2* protein (arrow head). **c** Immunoblot analysis of *His-Tol2* expression with anti-His antibody. The same protein samples as in (**b**) were loaded except that only ~ 6 ng purified *His-Tol2* was used for detection by western blot

We examined the expression of both N- and C-terminally tagged *Tol2* variants in *E. coli*. The C-terminally tagged version gave predominantly the full-length form with multiple, smaller protein products. In contrast, the amino-terminally tagged *Tol2* (Fig. 2a) yielded a single protein product at the predicted size (~74 kD) when purified from *E. coli* (Fig. 2b, c). This *His-Tol2* protein was used in all subsequent studies unless noted otherwise.

### Recombinant *Tol2* protein is a fully functional *hAT* transposase in vivo

**Fig. 3:**
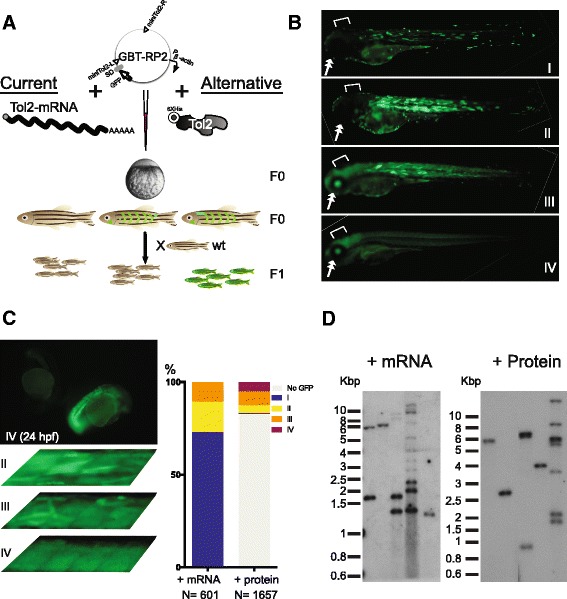
*His-Tol2* is a fully functional transposase in vivo. **a** Diagram showing microinjection of zebrafish embryo at one-cell stage to generate transgenic animals. GBT-RP2 plasmid containing GFP-reporter gene trap was co-injected with either *Tol2* mRNA or *His-Tol2* protein. Mosaic somatic GFP signals were seen in F0 injected fish. F0 embryos were raised to adult and outcrossed to obtain F1 generation. Ubiquitous GFP would be detected in F1 fish if the integration was due to a gene trap event and was passed through the germline. Molecular analysis of transposon insertion numbers and genomic locations was carried out on tissue from F1 generation animals. P_β_-actin: β-actin promoter; SD: splice donor. **b** Representative images of F0 embryos demonstrating a catalog of GFP-positive somatic patterns that could be observed at 3 dpf from microinjections of different methods. Notice the signal difference in eyes (double arrow) and brain regions (open end arrow) for each category. With vector-only injections, notice only Cat. I and II patterns were observed. **c** A small number of *His-Tol2* protein injected embryos showing ubiquitous whole body green fluorescence as early as 24 hpf. Those fish generally displayed uniform GFP expression throughout the body, with few uneven myotome GFP stripes at 3 dpf (Cat. IV). Also shown are 3 dpf injected fish somites at higher magnification, demonstrating the difference in uniformity of GFP signals in tissues from various injection categories. Injection GFP pattern distribution from typical RNA or protein mediated injection was shown. **d** GFP-positive F1 fish from either founder family generated by mRNA injection or *His-Tol2* injection were subjected to fin-clip and Southern blot analysis with a GFP probe. Each lane represents one individual F1 adult fish. Southern blots from 5 adult fish selected from random founder family are shown for each method. See also Additional file 1

Transposon tools are often used as a two-component system in gene discovery and gene delivery applications (Fig. 3a). One component is donor DNA containing a genetic cargo of interest flanked by transposon terminal sequences, and the other component is a transposase source. In previously described vertebrate applications, transposase was provided by either a DNA-encoding plasmid or in vitro transcribed transposase-encoding mRNA. To determine if purified recombinant *His-Tol2* protein is fully functional and robust enough to be an alternative transposase source for practical genetic applications, we tested *His-Tol2* function in vivo using this two-component system in both somatic and germline gene transfer assays.

For gene transfer testing in somatic cells, we used a high stringency transposition test system in which insertions of a specialized *miniTol2* element, GBT-RP2 [26], in transcriptionally competent genomic loci result in enhanced Green Fluorescent Protein (GFP) expression in the resulting animals. The GBT-RP2 element was based on the 3′ gene trap derived from previously described gene-breaking transposon (GBT) vectors [27, 28]. Transposon DNA was co-injected with either *Tol2* mRNA or *His-Tol2* protein into one-cell zebrafish embryos (Fig. 3a). The F0 somatic GFP patterns of injected embryos generally can be characterized into different categories based on signal uniformity at 3 days post-fertilization (dpf): a category I pattern is characterized by embryos showing minimal GFP signals, usually only a couple of bright stripes in the myotomes; a category II pattern is characterized by large areas of the fish body with GFP signals, but with little or no GFP-positive signals in the eyes or brain region; and a category III pattern is characterized by whole body green fluorescence, including the brain and eyes (Fig. 3b).

Background, non-transposase mediated gene transfer was measured by the injection of the RP2 vector without transposase, resulting in sporadic GFP signals seen in myotomal tissues of some F0 fish by 3 dpf. Usually, the majority of the background GFP-positive fish displayed category I patterns, and very few displayed category II patterns (Fig. 3b).

When transposase was provided by *Tol2* mRNA, the overall somatic GFP signals were notably increased, especially with a higher number of embryos exhibiting the category III pattern, which was a very rare observation without the co-addition of transposase (Fig. 3b, c).

With *His-Tol2* protein, the majority of the injected fish did not display any GFP signal. Among the GFP-positive F0 embryos, GFP patterns representing all three categories were noted. However, the number of category I animals, which were less likely to carry transposon-mediated germ-line integration, was much lower than the GFP-positive category II or category III (Fig. 3b, c). We also conducted multi-generational testing for germline gene transfer rate testing. F0 fish displaying category III somatic patterning from either mRNA or *His-Tol2* transposase resulted in a similar gene trapping rate and low mosaicism (Table 1).

**Table 1:** *His-Tol2* and *Tol2* mRNA as sources of transposase in vivo

Somatic F0 pattern	*Tol2* form	Germline trapping and expression frequency	Average F0 germline mosaicism
Cat II.	mRNA	65 % (13/20)	27 %
Cat III.	mRNA	88 % (35/40)	39 %
Cat III.	Protein	76 % (13/17)	39 %
Cat IV.	Protein	90 % (9/10)	51 %

Analyses of somatic and germline transmission of reporter genes are shown. Fish injected with either mRNA or *His-Tol2* were characterized by different F0 somatic patterns and assessed for corresponding germline trapping frequency, estimated as the percentage of F0 fish producing GFP-positive offspring. The number of fish screened is shown in parentheses. The F0 germline mosaicism rate was determined by the percentage of GFP-positive F1 offspring from a founder outcross. The average mosaicism rate for founder fish is listed from each different category

Interestingly, an additional pattern found only in protein-injected embryos was observed. A small portion of these injected animals (~5–10 %) exhibited ubiquitous GFP expression as early as 24 h after injection (Fig. 3c), and the signals were very uniform throughout the body at 3 dpf (Fig. 3c). Such F0 embryos were designated category IV (Table 1). Zebrafish embryos undergo rapid cell divisions every 20 min during the early development and reach ~ 1000 cells by 3 h post fertilization [29]. Thus the timing of transposition potentially plays a critical role in determining the mosaicism of resulting animals. We hypothesized that the uniform GFP signals of the category IV fish could be the result of very early integrations of the GFP reporter gene into the genome. Indeed, a higher percentage of germline transmission was obtained from such F0 fish (Table 1). More importantly, the average F0 germline mosaicism was much reduced for category IV fish (Table 1). Our observations of *His-Tol2* mediated injection suggest that even though transposition events were rarer under current condition than when compared to mRNA as a source of transposase, protein injection can “jump start” integration inside the early zebrafish embryo.

An important additional measurement of the germline gene transfer rate is copy number. Transposon copy number was estimated by Southern blot analysis on individual fish (Fig. 3d). An average of ~ 4 unique transposon insertions per haploid F0 founder germline were observed (Additional file 1). The fact that category IV (Additional file 1, family 1,2,3,5,6 and 8) did not contain more transposon insertions than in other groups suggests that the uniform fluorescence is most likely due to earlier incorporation of the reporter gene into the embryo genome.

Transposase-mediated insertion can be distinguished from other insertion mechanisms by the target site duplications flanking the transposon sequences. We sequenced the insertion sites of the *His-Tol2* protein-mediated RP2 transposon insertions, and the *hAT* signature 8-bp genomic duplications were present in all checked cases (Table 2). We conclude that *His-Tol2* protein is a fully functional *hAT* transposase in both somatic and germline lineages in vivo.

**Table 2:** Transposase-mediated germline integration

Site	Sequence	Chr.	Refseq
**1**	aaatatttac**caagcaac**-5′-Tol2-3′-**caagcaac**acgttcagtg	8	Intron of gfra2
**2**	ataatttcct**cttatttg**-5′-Tol2-3′-**cttatttg**catgtcagat	13	Intergenic
**3**	cgcatgctaa**cttataga**-5′-Tol2-3′-**cttataga**ggaggtgccc	8	Exon of wu: fb79a07
**4**	aaacgttcct**cctaacac**-5′-Tol2-3′-**cctaacac**agttagatgg	3	Intergenic
**5**	caacacatga**ctcgttgg**-5′-Tol2-3′-**ctcgttgg**ccatatgcta	15	Intergenic
**6**	gggaatatgt**gttattaa**-5′-Tol2-3′-**gttattaa**ctgcgtccca	4	Repetitive sequences
**7**	agctgtctct**tctgtgtc**-5′-Tol2-3′-**tctgtgtc**attcagtctc	3	Intron of LOC557901
**8**	tgtcagagat**ctaggtca**-5′-Tol2-3′-**ctaggtc**agatggaggaa	25	Intergenic

*Tol2* insertion sites generated by *His-Tol2* protein co-injection were cloned from F1 transgenic fish. Examples of 8-bp genomic sequence duplication (in bold) were shown with insertion loci information

### Recombinant *Tol2* protein can mediate concerted target joining in vitro

**Fig. 4:**
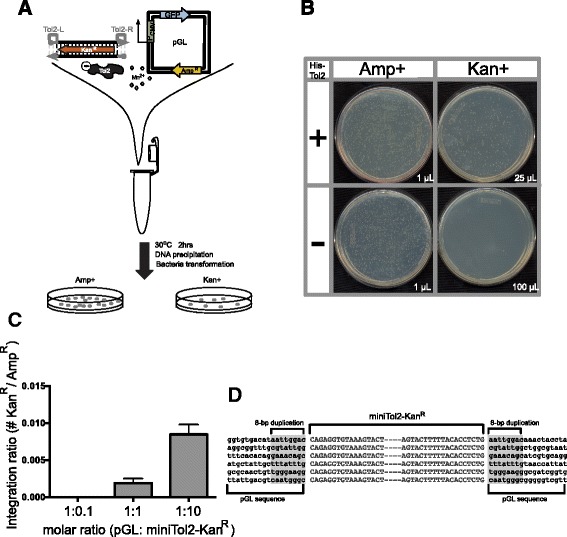
*Tol2* in vitro integration assay. **a** Scheme of in vitro integration assay. **b** One example *miniTol2-Kan* ^*R*^ integration, revealing different kanamycin-resistant colony recovery from assays with versus without *His-Tol2* protein. The amount of transformed bacteria spread on each plate is indicated. **c** *Tol2*-mediated *miniTol2-Kan* ^*R*^ integration. The amount of target plasmid (pGL) was kept at 0.5 pMol, while PCR fragments were mixed at three different ratios. The ratio of colony numbers (Additional file 3) recovered from LB-Kan^+^ to LB-Amp^+^ was used as an indicator of integration activity. Bars represent mean values ± SEM from three independent experiments. **d** Plasmids isolated from colonies on LB-Kan^+^ plate were sequenced at both junctions of *miniTol2-Kan* ^*R*^ insertion. 8-bp target plasmid duplications were indicated. Six examples of the integration junction are shown here

**Fig. 5:**
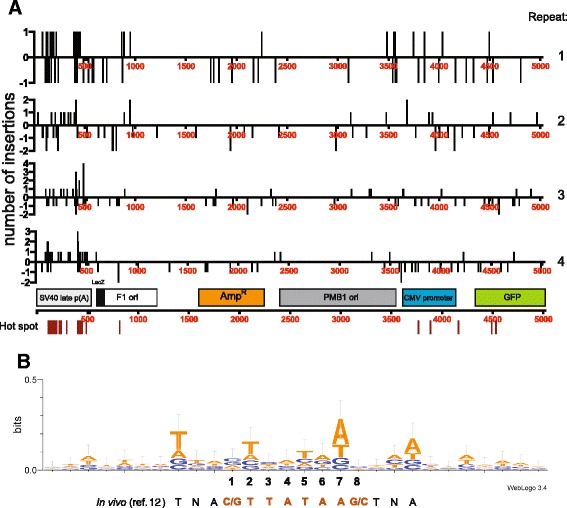
*miniTol2* insertion sites and consensus sequence. **a** *Tol2*-mediated insertions from four independent experiments were mapped to target plasmid (pGL) for *miniTol2-Kan* ^*R*^ (*n* = 266). Y-axis indicates the number of insertion events and orientation (positive: sense orientation; negative: anti-sense orientation) Major features of the pGL vector were also indicated. Red lines below the sequences indicate insertion “hot spots”, defined as the same locations that are discovered in more than one experiment, regardless of the insertion orientation or locations in one experiment that have transposons inserted in both directions (also see Additional file 5). **b** *Tol2* integration site motif analyzed by WebLog (version 3.0) (*n* = 266) and aligned to a previous indentified weak AT-rich consensus in vivo [12]

We continued to explore whether this recombinant *Tol2* protein is functional in vitro, focusing on the target joining step by directly assaying the ability of *His-Tol2* to join both ends of a DNA segment flanked by *Tol2* end sequences to a target plasmid (Fig. 4a). *Tol2* terminal sequences consisting of 261 bp from the left arm and 192 bp from the right arm (*miniTol2*) are sufficient transposon end sequences for efficient *Tol2* transposition in vivo [13, 14]. We used a PCR-generated *Kan*^*R*^ gene flanked by these *Tol2* ends (Additional file 2), *miniTol2-Kan*^*R*^ together with *His-Tol2* protein to assess target joining in vitro. After incubation of blunt end *miniTol2-Kan*^*R*^ with an ampicillin resistant target plasmid (pGL) in the presence of *His-Tol2* protein and Mn^2+^ ions, DNA was precipitated, transformed into *E. coli*, and plated on LB-Amp^+^ plates to determine the total number of target plasmids and on LB-Kan^+^ plates to identify integration products (Fig. 4a, b). The number of *Tol2* integrants increased as the number of transposons in the reaction increased (Fig. 4c, Additional file 3), and we detected up to 0.8 % of integration ratio among the conditions tested. We sequenced large numbers of integrants obtained in multiple experiments and found that the 8-bp host duplications at the insertion site characteristic of *hAT* transposition occurred in 86 % of insertions (Total *N* = 333) (Fig. 4d). Thus *His-Tol2* integration in vitro recapitulates *Tol2* integration in vivo. The distribution of recovered insertions in the target plasmid was non-random with *miniTol2* element insertion biased towards the AT-rich (AT content 64 %) SV40 polyA segment of the target vector (Fig. 5a; see also below). We also noticed that *Tol2* protein was able to excise a *miniTol2*-flanking fragment out of a plasmid donor (data not shown), indicating *His-Tol2* is fully competent gene transfer vector in vitro as well as in vivo.

### Target site preference of *Tol2* is conserved using a simplified target plasmid system

As described above, the *miniTol2-Kan*^*R*^-insertions generated in vitro were not randomly distributed on the target plasmid, and they clustered at the SV40 poly (A) rich region. Analysis of these patterns by Monte Carlo simulation demonstrated that the bias was highly significant (p-value by simulation < 0.0001, see Methods) in each of four independent experiments (Fig. 5a; Additional file 4 and Additional file 5). This observed preference in vitro is in agreement with prior work showing that *Tol2* has a preference for AT-rich regions in vivo*.* More importantly*,* the in vivo observed integration signature, TNA**(C/G)TTATAA(G/C)**TNA (bold: insertion site) [12], was similar to the findings reported here using the plasmid insertion system (Fig. 5b). This observation suggests a primary sequence of the target site is likely to play a critical role in *Tol2*/*hAT* integration site selection.

## Discussion

In the past decade, the *Tol2*-based transposition system has become a versatile tool for gene delivery in commonly used model organisms, especially in zebrafish, which has seen many cases of successful genetic screening with various highly efficient Tol2 systems [30, 31]. Recently, *Tol2* transposon tools have been applied to pioneer gene transfer in novel biological systems, such as haplochromine cichlid [32, 33] and African killifish [33]; or as an alternative to viral vectors showing promise in engineered T-cell therapy [34]. Here we report the purification of recombinant *Tol2 hAT* transposase from *E. coli* and demonstrate that it is fully functional in vivo and in vitro. A functional recombinant *Tol2* protein will be a useful addition to the *Tol2*-based genome engineering kit. Researchers will have more choices over what form of transposase will work best for the organisms/cells of their interest. Transposase provided as readily made protein, in some systems, may be advantageous over an mRNA-based delivery method. For example, when the translation of the *Tol2* mRNA is not efficient or optimized for the intended heterologous system. And even in zebrafish where mRNA is an effective method for Tol2 delivery, we noted advantages of using the functional Tol2 transposase protein.

We observed robust somatic and germline transposition in zebrafish using *E.coli* -purified *Tol2* transposase. Our experiments suggest using *His-Tol2* may be advantageous in generating low mosaic transgenic animals with higher germline transmission rate. Transposition using a protein source of transposase may “jumpstart” transposition as it avoids possible delays from transcription, translation and assembly of a functional transpososome containing transposase multimers when transposase is supplied in a ready form. Transposase multimers are the active form of transposase in other studied systems [7, 35, 36]. Static light scattering analysis has shown that purified *Tol2* is a dimer (A. Voth and F. Dyda, personal communication). This recombinant *Tol2* protein is an excellent tool for future experimental methods for gene delivery. As documented using this initial system, the percentage of GFP-positive F0 embryos was lower using protein as a transposase source when compared to the well-established mRNA co-injection methods,. However, the current protein co-injection method has yet to be fully optimized, such as experimentally establishing the optimal protein to donor plasmid ratio.

Functional *Tol2* transposase protein is also of use for studying transposon biology science, enabling experimental testing of this *hAT* element both in vivo and in vitro. Such work will shed more light on our understanding of how this and related transposons work in the future. In this paper, we demonstrated that Tol2 protein could be used in an assay to probe the integration site preference for the transposon and identified a weak consensus that are largely conserved between in vivo chromosomes and a cell-free target plasmid. Two other commonly used transposases for genome engineering, *Sleeping Beauty* and *piggyBac* use obligate TA and TTAA core consensus targeting sequence respectively [37, 38]. In contrast, *Tol2* target site selection is still considered promiscuous on the level of primary DNA sequence [39]. The preference noticed towards AT-rich DNA context could also reflect a preference towards integration into regions of higher DNA flexiblity as suggested previously [40–42].

## Conclusions

We presented here a fully functional recombinant transposase protein, both in vitro and in vivo, for the popular vertebrate gene transfer transposon *Tol2*. Our initial work in zebrafish indicated that *His-Tol2* has a high potential for genome engineering applications. *His-Tol2* can also serve as a tool for probing transposon biology in vitro and help confirm an integration site consensus shared between in vivo and cell-free systems.

## Methods

### Ethics statement

Zebrafish larvae were raised within the Mayo Clinic’s Zebrafish Facility with adherence to the NIH Guide for the Care and Use of Laboratory Animals and approval by Mayo Clinic’s Institutional Animal Care and Use Committee.

### Important constructs

Details of how important constructs were made are included in Additional file 6: Supplemental Experimental Procedures.

### HeLa cell transfection assays

Transfection of HeLa cells with a Zeocin resistant vector (pTol2miniZeo) with either control vector pCMV-GFP or vectors providing *Tol2* transposase (pCMV-Tol2-M, pCMV-hTol2-M or pCMV-hTol2-L) was following the same protocol as described in [21]. Zeocin-resistant colony forming assay was conducted as in [21] as well.

### Zebrafish somatic transposition assays

To compare transposition of a GFP-reporter mediated by tagged vs. untagged *Tol2* mRNA (Fig. 1d), zebrafish embryos were injected at one-cell stage with a mixture of 25 pg pTol2-S2EF1α-GM2 (pDB591, [13]) plasmid DNA with 25 pg or 100 pg T3TS-Tol2-M (*Tol2*), T3TS-His-Tol2, or T3TS-His-Tol2 RNA (Fig. 1d) respectively. Surviving normal-looking embryos were scored for GFP fluorescence at 3 dpf.

### Protein expression and immunoblotting

Small scale detection of protein expression was conducted according to Qiagen recommended protocol. Detail procedures are included in Additional file 6: Supplemental Experimental Procedures. *His-Tol2* protein expression was detected by penta-His antibody (Qiagen) following the recommended western protocol. An adapted protocol [43] was used to generate large-scale nuclease-free protein fractions.

### Zebrafish one-cell GBT microinjection

One-cell stage zebrafish microinjection was performed as described [44]. The injected amount of reagents were 25 pg GBT-RP2 plasmid co-injected with 25 pg *T3TS-Tol2* mRNA [13]; or co-injected with ~ 0.2 ng *His-Tol2* protein.

### Southern blot

Genomic DNA isolation and Southern blot hybridization was conducted as previously described [28]. Tail fins from individual GFP-positive F1 fish generated by co-injection of *Tol2* mRNA or *His-Tol2* protein at F0 were clipped and genomic DNA was isolated followed by digestion with BamHI and BglII for Southern blotting.

### Molecular analysis of genomic integration sites

Cloning of insertion site in F1 individual fish was conducted through adapted inverse and linker-mediated PCR (LM-PCR) [44]. Information of primer sequences and PCR conditions are included in Additional file 6: Supplemental Experimental Procedures.

### In vitro integration assay

Blunt end *miniTol2-Kan*^*R*^ fragments were generated by PCR with platinum Pfx DNA polymerase (Invitrogen) with forward primer Tol2-mini5′ (5′- CAGAGGTGTAAAGTACTTGA-3′), reverse primer Tol2-mini3′ (5′- CAGAGGTGTAAAAAGTACTC-3′) and a template (pTol2miniKan(-)amp). PCR product was treated with DpnI to get rid of the template before purified by QIAquick PCR purification kit (Qiagen). Freshly prepared plasmids and PCR products were used in the assay. In a 20 μL reaction, 0.5 pMol plasmid pGL, corresponding PCR fragments and 27 pMol *His-Tol2* (~2 μg) were mixed in MOPS buffer (25 mM MOPS, pH 7.0; 1 mM MnCl_2_; 50 mM NaCl; 5 % glycerol; 2 mM DTT; 100 ng/ul BSA). The reaction was incubated for 2 h at 30 °C. DNA was phenol-chloroform extracted using Maxtract High Density columns (Qiagen) and resuspended in 5 μL TE buffer. 1 μL or 5 μL DNA was used to transform Top10 competent cells (Invitrogen). The number of colonies grew on Amp-resistant plates was used to calculate the total number recovered plasmids, and the number of colonies that grew on Kan-resistant plates was used to calculate the number of plasmid with Kan^R^ integration. MOPS buffer with MgCl_2_ or no added cations were tested as well, and the integration activity was modestly higher with MnCl_2_.

### Insertion site distribution statistics

The simulation distributions of the number of insertions over the target features was done in R. We assume that every feature gets a number of distributions proportional to its size in bp. We simulated 75 counts with the probability of a count falling into a feature being the length of that feature divided by the length of all features types. We then ran this simulation 10,000 times. To check where the experimental data lie with respect to the simulated data, we determined how often the simulated counts are bigger or smaller than the experimental data.

## Additional files

Additional file 1: Estimation of transposon insertion numbers by Southern analysis. Individual GFP-positive F1 fish from random selected F0 founder families were fin-clipped for gDNA isolation. Southern blotting with a GFP probe was conducted for each fish gDNA and the number of clear-labeled Southern bands was recorded as estimation of transposon insertions. Total number of unique Southern bands was used to estimate total transposon insertions for each F0 founder family. An average insertion number over all the families analyzed was calculated for both injection methods. (DOCX 91 kb) Additional file 2:
*miniTol2* sequences of the in vitro assays. DNA sequences of the left arm and right arm of *miniTol2* flanking the Kan^R^ used in the in vitro integration assays. (DOCX 55 kb) Additional file 3: Colony counting numbers for in vitro integration assays. Colony numbers counted for the three independent experiments in Fig. 4c of different target plasmid and PCR insert ratios. (DOCX 49 kb) Additional file 4:
*P*-value by simulation. For each independent repeat of *miniTol2* mediated Kan^R^ insertion distribution, p-value was generated by simulation (see Methods) for different features on the target plasmid. (DOCX 61 kb) Additional file 5: Insertion “hot spots” for *miniTol2*. “Hot spots” were arranged according to the insertion loci along the target plasmid. 8-bp target duplication and flanking sequnces were shown. Forward (F) or reverse (R) insertion copies were counted. Repeats coverage indicate the “hot spot” indentified by how many independent experiments. (XLSX 45 kb) Additional file 6: Supplemental Experimental Procedures. (DOCX 131 kb)

## Abbreviations

Ac/Ds: Activator/Dissociation
TEs: transposable elements
TIRs: terminal inverted repeats
GBT: gene-breaking transposon
dpf: days post-fertilization
*Kan*^*R*^: kanamycin resistance
