# Supplementary material for: Active recombinant Tol2 transposase for gene transfer and gene discovery applications
Source: Mob DNA. 2016 Mar 31;7:6. doi: 10.1186/s13100-016-0062-z (PMC4818426; doi:10.1186/s13100-016-0062-z)
Supplement: Additional file 1: — Estimation of transposon insertion numbers by Southern analysis. Individual GFP-positive F1 fish from random selected F0 founder families were fin-clipped for gDNA isolation. Southern blotting with a GFP probe was conducted for each fish gDNA and the number of clear-labeled Southern bands was recorded as estimation of transposon insertions. Total number of unique Southern bands was used to estimate total transposon insertions for each F0 founder family. An average insertion number over all the families analyzed was calculated for both injection methods. (DOCX 91 kb) [file 13100_2016_62_MOESM1_ESM.docx]

**Additional file 1. Estimation of transposon insertion numbers by Southern analysis.**

Individual GFP-positive F1 fish from random selected F0 founder families were fin-clipped for gDNA isolation. Southern blotting with a GFP probe was conducted for each fish gDNA and the number of clear-labeled Southern bands was recorded as estimation of transposon insertions. Total number of unique Southern bands was used to estimate total transposon insertions for each F0 founder family. An average insertion number over all the families analyzed was calculated for both injection methods.

|  | F0 family ID | F1 fish ID | # Est. insertions | # Est. unique insertions/family |
| --- | --- | --- | --- | --- |
| + mRNA | #82 | f4 | 4 |  |
|  |  | f5 | 2 |  |
|  |  |  |  | 4 |
|  | #93 | f5 | 2 |  |
|  |  |  |  | 2 |
|  | #112 | f3 | 1 |  |
|  |  | f4 | 1 |  |
|  |  |  |  | 2 |
|  | #282 | f2 | 1 |  |
|  |  |  |  | 1 |
|  | #173 | f1 | 1 |  |
|  |  |  |  | 1 |
|  | #192 | f2 | 11 |  |
|  |  | f4 | 8 |  |
|  |  |  |  | 11 |
|  |  |  | Ave. | **3.5** |
|  |  |  |  |  |
| + *His-Tol2* | #1 | f1 | 3 |  |
|  |  | f2 | 8 |  |
|  |  | f4 | 3 |  |
|  |  | f6 | 2 |  |
|  |  | f7 | 1 |  |
|  |  | f8 | 11 |  |
|  |  |  |  | 11 |
|  | #2 | f1 | 1 |  |
|  |  | f2 | 1 |  |
|  |  | f5 | 1 |  |
|  |  |  |  | 1 |
|  | #3 | f4 | 1 |  |
|  |  |  |  | 1 |
|  | #5 | f2 | 2 |  |
|  |  | f3 | 1 |  |
|  |  |  |  | 2 |
|  | #6 | f2 | 3 |  |
|  |  | f3 | 1 |  |
|  |  | f6 | 4 |  |
|  |  | f7 | 1 |  |
|  |  |  |  | 6 |
|  | #8 | f2 | 1 |  |
|  |  | f3 | 3 |  |
|  |  |  |  | 3 |
|  | #15 | f2 | 1 |  |
|  |  |  |  | 1 |
|  | #17 | f1 | 2 |  |
|  |  | f2 | 1 |  |
|  |  |  |  | 2 |
|  | #18 | f1 | 1 |  |
|  |  | f3 | 1 |  |
|  |  |  |  | 1 |
|  |  |  | Ave | **3.1** |
