# Supplementary material for: Active recombinant Tol2 transposase for gene transfer and gene discovery applications
Source: Mob DNA. 2016 Mar 31;7:6. doi: 10.1186/s13100-016-0062-z (PMC4818426; doi:10.1186/s13100-016-0062-z)
Supplement: Additional file 2: — miniTol2 sequences of the in vitro assays. DNA sequences of the left arm and right arm of miniTol2 flanking the KanR used in the in vitro integration assays. (DOCX 55 kb) [file 13100_2016_62_MOESM2_ESM.docx]

**Additional file 2. *miniTol2* sequences of the *in vitro* assays.**

DNA sequences of the left arm and right arm of *miniTol2* flanking the Kan^R^ used in the *in vitro* integration assays.

>*miniTol2-L* arm

CAGAGGTGTAAAGTACTTGAGTAATTTTACTTGATTACTGTACTTAAGTATTATTTTTGGGGATTTTTACTTTACTTGAGTACAATTAAAAATCAATACTTTTACTTTTACTTAATTACATTTTTTTAGAAAAAAAAGTACTTTTTACTCCTTACAATTTTATTTACAGTCAAAAAGTACTTATTTTTTGGAGATCACTTCATTCTATTTTCCCTTGCTATTACCAAACCAATTGAATTGCGCTGATGCCCAGTTTAATTT

>*miniTol2-R* arm

GAATCTCTAGTTTTCTTTCTTGCTTTTACTTTTACTTCCTTAATACTCAAGTACAATTTTAATGGAGTACTTTTTTACTTTTACTCAAGTAAGATTCTAGCCAGATACTTTTACTTTTAATTGAGTAAAATTTTCCCTAAGTACTTGTACTTTCACTTGAGTAAAATTTTTGAGTACTTTTTACACCTCTG
