# Supplementary material for: Active recombinant Tol2 transposase for gene transfer and gene discovery applications
Source: Mob DNA. 2016 Mar 31;7:6. doi: 10.1186/s13100-016-0062-z (PMC4818426; doi:10.1186/s13100-016-0062-z)
Supplement: Additional file 3: — Colony counting numbers for in vitro integration assays. Colony numbers counted for the three independent experiments in Fig. 4c of different target plasmid and PCR insert ratios. (DOCX 49 kb) [file 13100_2016_62_MOESM3_ESM.docx]

**Additional file 3. Colony counting numbers for *in vitro* integration assays**

Colony numbers counted for the three independent experiments in Fig. 4C of different target plasmid and PCR insert ratios.

| pGL:miniTol2-Kan^R^ | # Recovered colonies | Transformed bacteria (uL) | 1 | 2 | 3 |
| --- | --- | --- | --- | --- | --- |
| 10:1 | Amp+ | 10 | 1149 | 1445 | 118 |
|  | Kan+ | 250 | 0 | 0 | 0 |
| 1:1 | Amp+ | 10 | 402 | 216 | 588 |
|  | Kan+ | 250 | 31 | 9 | 15 |
| 1:10 | Amp+ | 10 | 201 | 1066 | 464 |
|  | Kan+ | 250 | 40 | 287 | 99 |
