# Supplementary material for: Active recombinant Tol2 transposase for gene transfer and gene discovery applications
Source: Mob DNA. 2016 Mar 31;7:6. doi: 10.1186/s13100-016-0062-z (PMC4818426; doi:10.1186/s13100-016-0062-z)
Supplement: Additional file 4: — P-value by simulation. For each independent repeat of miniTol2 mediated KanR insertion distribution, p-value was generated by simulation (see Methods) for different features on the target plasmid. (DOCX 61 kb) [file 13100_2016_62_MOESM4_ESM.docx]

**Additional file 4. P-value by simulation.**

|  |  | *miniTol2* Repeats | | | |
| --- | --- | --- | --- | --- | --- |
|  |  | **p-value** | | | |
| Features | **Size (bp)** | **1** | **2** | **3** | **4** |
| LacO | 23 | 0.0004 | 0.2933 | 0.2933 | 0.0468 |
| LacZ a | 69 | 0.2724 | 0.274 | 0.0812 | 0.2724 |
| SV40 late p(A) | 520 | 0 | 0 | 0 | 0 |
| CMV promoter | 527 | 0.6790 | 0.2666 | 0.2666 | 0.3937 |
| F1 ori | 534 | 0.6894 | 0.0927 | 0.9630 | 0.9113 |
| Amp^R^ | 660 | 0.8829 | 0.9386 | 0.9386 | 0.6659 |
| GFP | 717 | 0.9654 | 0.9256 | 0.9256 | 0.3809 |
| pMB1 ori | 1160 | 0.9999 | 0.9978 | 1 | 1 |

For each independent repeat of *miniTol2* mediated Kan^R^ insertion distribution, p-value was generated by simulation (see Methods) for different features on the target plasmid.
