# Supplementary material for: Active recombinant Tol2 transposase for gene transfer and gene discovery applications
Source: Mob DNA. 2016 Mar 31;7:6. doi: 10.1186/s13100-016-0062-z (PMC4818426; doi:10.1186/s13100-016-0062-z)
Supplement: Additional file 6: — Supplemental Experimental Procedures. (DOCX 131 kb) [file 13100_2016_62_MOESM6_ESM.docx]

**Supplemental Experimental Procedures**

**Important constructs:**

*Tol2* mRNA variants: For *in vitro* transcription of transposase mRNA, all corresponding cDNA sequences were cloned into transcription vector pT3TS [1] . . The *Tol2-M* version is from plasmid pDB600 [2]. We generated longer versions based on published literature [3,4]. *Tol2-L* (pDB598) was PCR- amplified using primers 5’-GCTGGATCCACCatgttcattggtcctttggaagtgacgtcatgtcacatctattaccacaatg

-3’ and 5’-CAGACTAGTctactcaaagttgtaaaacc-3’, cloned into pCRIItopo (Invitrogen) to generate pDB592, and then subcloned as BamHI-SpeI fragment into pT3TS transcription vector [1] and digested with BglII-SpeI to generate pDB598.

Tagged-*Tol2* mRNA: For *in vitro* transcription of transposase mRNA, all corresponding cDNA sequences were cloned into transcription vector pT3TS. *Tol2* transposase with an N-terminal 6XHis tag (His-Tol2; pDB747) was generated by PCR amplifying *Tol2* fragment with forward primer 5’-gcaGgaTCCACCatgCATCATCATCATCATCATgaggaagtatgtgattcat – 3’ and reverse primer 5’-CAGACTAGTctactcaaagttgtaaaacc-3’ using pDB600 [2] as a template. The PCR fragment was cloned into pCRIItopo vector (Invitrogen) to generate pDB683, and then subcloned into BglII-SpeI digested pT3TS transcription vector as BglII-SpeI fragment to generate pDB747. Tol2 transposase with a C-terminal 6XHis tag (Tol2-His; pDB748) was amplified using primers 5’-GCTGGATCCACCatggaggaagtatgtgattc-3’ and 5’-gtcactAGTctaATGATGATGATGATGATGctcaaagttgtaaaacct-3’, then cloned into pJET1 PCR Cloning vector (Fermentas) to generate pDB684, and then subcloned into BglII-SpeI digested pT3TS transcription vector as a BamHI-SpeI fragment to generate pDB748. Both pDB747 and pDB748 were linearized with XbaI transcribed with T3 RNA polymerase to produce *Tol2* RNA.

*In vitro* transposition in HeLa cells: Cargo plasmid pTol2miniZeo, control plasmid pCMV-GFP, transposase vectors providing *Tol2-M* (pCMV-Tol2), and codon –optimized *hTol2-M* (pCMV-iTol2) were described in [5].

Recombinant Tol2 protein expression: For *E.coli* expression and purification of Tol2 protein, a His(6X)-Tol2 ORF was PCR-generated from template pDB747 (above) with forward primer: 5’-ATGCATCATCATCATCATCATGAG-3’ and reverse primer: 5’- CTACTCAAAGTTGTAAAACC-3’. The PCR fragment was cloned into pET-21a (Novagen) through XbaI and BamHI sites to generate pET21-His-Tol2.

Details on other plasmid constructs used are available upon request.

**Protein expression**

Overnight transformed BL21-AI (Invitrogen) cultures were diluted at ~ 1:100 to inoculate fresh LB medium containing the appropriate antibiotic to an OD_600_ of 0.5-0.7 at 30°C. Just prior to induction, a 50 μL sample of culture was removed and mixed with 50 μL 2XSDS-PAGE sample buffer (uninduced control). His-Tol2 expression was induced by 1mM IPTG and 0.2% L-arabinose for ~ 16 hrs at 16°C. Prior to harvesting the cells, a 50 uL sample was removed and mixed with 50 μL 2XSDS-PAGE sample buffer (induced total crude lysate). Cells were harvest by centrifuge at 4000g for 20 minutes and pellets could be stored at -80°C for future purification. For protein expression confirmation (Figure 2B), cell pellet from 1 mL culture was sonicated and the cell lysate was centrifuged at 10,000g at 4°C for 30 min. 50 μL of supernatant was mixed with 50 μL 2XSDS-PAGE sample buffer (soluble protein). The pellet was resuspended in lysate buffer back to 1 mL and prepared for SDS sample (insoluble protein). 15 μL SDS-sample of each preparation was used for SDS-PAGE analysis on 4-15% gradient gel (Bio-Rad) in Figure 2B. Proteins were visualized by GelCode Blue Stain (Pierce Biotechnology).

**LM-PCR**

Genomic DNA was isolated from individual tail fin clipps using the protocol described above. LM-PCR was performed using 500 ng of gDNA for the initial restriction enzyme digestion with AvrII, NheI, SpeI and XbaI (NEB) in a 10 µl reaction volume over night at 37°C. The enzyme reaction was terminated using heat-inactivation at 65°C for 20 min. 5 µl from the initial enzyme digest was subjected to blinkerette linker ligation using blinkerette-XbaI linkers. To make the blinkerette-XbaI linker, the following oligonucleotide sequences were annealed together at 80°C for 5 min and cooled down to room temperature slowly:

primerette-long 5’-CCTCCACTACGACTCACTGAAGGGCAAGCAGTCCTAACAACCATG-3’ and blinkXbaI (5’P)-CTAGCATGGTTGTTAGGACTGCTTGC(dideoxy)-3’.

Linker ligations were performed overnight at 16°C in a 10 µl reaction volume using T4 DNA ligase (NEB). 2 µl from a 1:100 dilution of the ligation mixture in 10 mM Tris-Cl (pH 8.0) was used for primary PCR using the following primers:

Left side: primerette-short, 5’-cctccactacgactcactgaagggc-3’ and Tol2-ITR(L)-O1, 5’--AATTAAACTGGGCATCAGCGCAATT-3’. Right side: primerette-short and 5R-mRFP-P1, 5’--cgcccttggtcaccttcagctt -3’. PCR conditions for Biolase polymerase (Bioline) were according to the manufacturer’s instructions. Initial denaturing step of 95°C for 1 min; 20-cycles of denature at 95°C for 5 seconds, annealing at 70°C for 3 min and decrease 0.5°C per cycle; 10-cycles of denature at 95°C for 5 seconds, annealing at 60°C for 3 min; a final extension at 70°C for 10 min. 1 µl of the diluted sample (1:100) of the first PCR product was used as template for the secondary PCR. Primers required for the secondary PCR are:

Left side: primerette-nested, 5’-GGGCAAGCAGTCCTAACAACCATG-3’ and Tol2-ITR(L)-O2 5’-- GCGCAATTCAATTGGTTTGGTAATAGC -3’. Right side: primerette-nested and 5R-mRFP-P2, 5’--CCTTGAAGCGCATGAACTCCTTGAT-3’.

PCR conditions: denaturing step of 95°C for 1 min; 5-cycles of denature at 95°C for 5 seconds, annealing at 65°C for 30 seconds and decrease 1°C per cycle and extension at 70°C for 3 min; 5-cycles of denature at 95°C for 5 min, annealing at 60°C for 30 seconds and extension at 70°C for 3 min; a final extension at 70°C for

10 min. PCR products were separated on a 1.5% agarose gel and individual bands were extracted using the QiaEx gel extraction kit (Qiagen) according to the manufacturer’s instructions for future subcloning and sequencing.

**Inverse-PCR**

4 µg gDNA was digested with the same set of restriction enzymes described in LM-PCR for 4-6 hrs at 37°C. 250 ng digested gDNA was subjected to ligation with T4 DNA ligase (NEB) at 4°C overnight. 2 µl from 1:10 dilution of ligation reaction was used for primary PCR. Primer sets used for Tol2 left TIR is Tol2-ITR(L)-O1 and 5R-GFP-P1 (5’- TGCATCACCTTCACCCTCTCCACT-3’); for right TIR is INV OPT 1 (5’- ATCCCTCACTCCCGTAGCTGTCCA-3’) and 5R-mRFP-P1 (above). 2 µl from 1 to 10 dilution of primary PCR was used for secondary PCR. Primer sets used for left TIR is 5R-GFP-P2 (5’- GGAAATTTGTGCCCATTAACATCACC-3’) and Tol2-ITR(L)-O2 (above); for right TIR is INV OPT 2 (5’- GCGAGCCAAACCTGTCTGGTGTA-3’) and 5R-mRFP-P2 (above).

PCR conditions: an initial denaturing step of 94°C for 2 min; 30-cycles of denature at 92°C for 30 seconds, annealing at 55°C for 30 seconds and extention at 70°C for 6 min; a final extension at 72°C for 10 min.

PCR products were separated on a 1% agarose gel and individual bands were extracted using the QiaEx gel extraction kit (Qiagen) according to the manufacturer’s instructions for future subcloning and sequencing.

**References:**

1. Hyatt TM, Ekker SC (1999) Vectors and techniques for ectopic gene expression in zebrafish. *Methods Cell Biol* 59: 117-126.

2. Balciunas D, Wangensteen KJ, Wilber A, Bell J, Geurts A, Sivasubbu S, Wang X, Hackett PB, Largaespada DA, McIvor RS, Ekker SC (2006) Harnessing a high cargo-capacity transposon for genetic applications in vertebrates. *PLoS Genet* 2: e169.

3. Koga A, Suzuki M, Maruyama Y, Tsutsumi M, Hori H (1999) Amino acid sequence of a putative transposase protein of the medaka fish transposable element Tol2 deduced from mRNA nucleotide sequences. *FEBS Lett* 461: 295-298.

4. Kawakami K, Shima A (1999) Identification of the Tol2 transposase of the medaka fish Oryzias latipes that catalyzes excision of a nonautonomous Tol2 element in zebrafish Danio rerio. *Gene* 240: 239-244.

5. Keng VW, Ryan BJ, Wangensteen KJ, Balciunas D, Schmedt C, Ekker SC, Largaespada DA (2009) Efficient transposition of Tol2 in the mouse germline. *Genetics* 183: 1565-1573.
